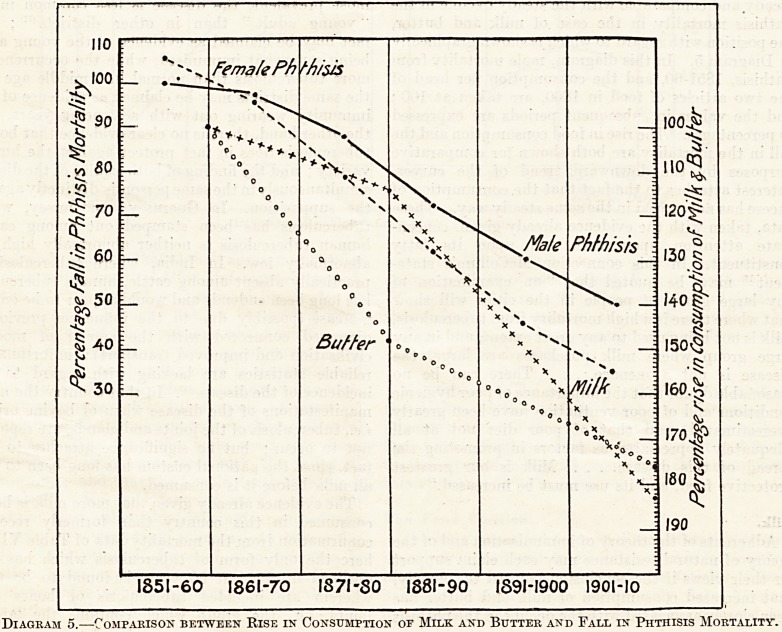# The Decline and Fall (?) of Tuberculosis.—III

**Published:** 1922-06

**Authors:** Edgar L. Collis

**Affiliations:** (Mansel Talbot Professor of Preventive Medicine, Welsh National School of Medicine)


### THE DECLINE AND FALL (?) OF TUBERCULOSIS.—III.

By EDGAR L. COLLIS, M.A., M.D., M.R.C.P. (Mansel Talbot Professor of Preventive Medicine, Welsh National School of Medicine.)

*(Concluded.)*

**Influence of Industry.**

Industrial work (apart from special risk associated with the inhalation of silica dust) has been recognised [12] to exert a marked influence upon the prevalence of phthisis; this position calls for consideration. Males may be taken, when compared with females, to represent the effect of industrial employment and females the effect of home environment, but in neither case are these two influences entirely restricted according to sex. Mortality data, shown graphically in Diagram 4, indicate that at early ages females suffer more than males, but that after adolescence is passed, with the introduction of occupational life, males go ahead and (as is brought out by comparing rural districts with county boroughs) go ahead most where industrial employment is most pronounced. The position seems to be that the more is the strain and stress in life to which any class is subjected, the more does that class succumb to tuberculosis. Greenwood has submitted the whole matter to close statistical study[13] and finds that the male death-rate from tuberculosis is highly correlated with the index of overcrowding; and that the female death-rate is not so correlated. He puts forward as a provisional explanation that "the industrial employment of men (as of women) lowers their resistance to tubercular infection; the principle focus of such infection is domestic; those exposed both to factory influence and unfavourable home environment succumb at a higher rate to tuberculosis."

The influence of industry upon women in war time and normally upon males, in no way controverts what has been already pointed out in reference to the importance of food. Those industrially employed expend more energy than those not so employed, and accordingly require not only a greater supply of food, but also of vitamins. Foods containing vitamins, especially fat-soluble A., are expensive and are exactly the foods which are most difficult for the industrial population to obtain in sufficient amount. The effect upon the general sickness-rate among workers of establishing factory canteens has been noted by Vernon[14]. Thus, in a Bristol factory the sickness-rate among the workers using the dining-room fell gradually until it eventually stood at half its former height.

DIAGRAM 4.—MORTALITY FROM PHTHISIS.

DEDUCTION.—The probable influence causative of the decline of tuberculosis has been a lessening of poverty; and the element in poverty concerned has been food. Evidence from the war period and from the influence of industrial occupation rather supports this view.

**The Food Position.**

The facts presented seem to direct attention to the question of food and to establish a case for inquiry into how the consumption of various articles of food has varied since 1850. Fortunately data bearing upon this question have recently been prepared by

Miss W. A. MacKenzie, Rothamsted Experimental Station, Harpenden, and the following table is taken from her research.

Table VII.—Consumption of the Principal Articles of Food in the United Kingdom in the Years 1860, 1880 and 1909-13.

| Commodities. | Total Consumption—Thousand tons. | | | | | | | | | Consumption per head per Week.—Lbs. | | |
|---|---|---|---|---|---|---|---|---|---|---|---|---|
| | 1860 | | | 1880 | | | 1909-13 | | | 1860. | 1880. | 1909–13. |
| | Impts. | Home Production | Total. | Impts. | Home Production | Total. | Impts. | Home Production | Total. | Pop. 28·7 mil. | Pop. 34·9 mil. | Pop. 45·2 mil. |
| Wheat (grain) ... ... | 1,070 | 3,070 | 4,140 | 3,300 | 2,070 | 5,370 | 5,876 | 824 | 6,700 | 6·20 | 6·60 | 6·40 |
| Meat and Bacon ... | 150 | 1,050 | 1,200 | 310 | 1,147 | 1,457 | 1,067 | 1,574 | 2,641 | 1·80 | 1·80 | 2·50 |
| Milk, fresh, min. gals. ... | — | 328 | 328 | — | 500 | 500 | — | 961 | 961 | pt. 1·75 | 2·20 | 3·20 |
| „ condensed... ... | — | — | — | — | — | — | 54 | — | 54 | — | — | 0·05 |
| Butter ... ... ... | 36 | 80 | 116 | 114 | 89·5 | 204 | 203 | 113 | 316 | 0·17 | 0·25 | 0·30 |
| Margarine ... ... | — | — | — | — | — | — | 58 | 59 | 117 | — | — | 0·10 |
| Cheese ... ... ... | 28 | 50 | 78 | 87 | 45 | 132 | 115 | 60 | 175 | 0·12 | 0·16 | 0·14 |
| Potatoes... ... ... | 28 | 4,500 | 4,528 | 121 | 4,500 | 4,621 | — | 4,183 | 4,138 | 6·80 | 5·70 | 4·00 |
| Rice ... ... ... | 17·5 | — | 17·5 | 218 | — | 218 | 303 | — | 303 | 0·03 | 0·30 | 0·29 |
| Tea ... ... ... | 34 | — | 34 | 71 | — | 71 | 133 | — | 133 | 0·05 | 0·09 | 0·13 |
| Sugar ... ... ... | 439 | — | 439 | 980 | — | 980 | 1,501 | — | 1,501 | 0·66 | 1·21 | 1·40 |
| Cocoa ... ... ... | 1·4 | — | 1·4 | 4·7 | — | 4·7 | 35 | — | 35 | 0·00 | 0·01 | 0·03 |
| Coffee ... ... ... | 16 | — | 16 | 14 | — | 14 | 12·7 | — | 12·7 | 0·02 | 0·02 | 0·01 |

Compiled by Miss W. A. Mackenzie.

The consumption of each article of food per head of population, with the exception of rice and potatoes, is found to have materially increased during the period under review ; but this increase has only been steady and comparable with the steady decline in the phthisis mortality in the case of milk and butter, the position with regard to which is shown graphically in Diagram 5. In this diagram, male mortality from phthisis, 1851-60, and the consumption per head of the two articles of food in 1860, are taken at 100 ; and the values in subsequent periods are expressed as percentages. The rise in food consumption and the fall in the mortality are both shown for comparative purposes by the downward trend of the curves. Interest attaches to the fact that the consumption of cheese has not moved in the same steady way. These data, taken with the evidence already given, concentrate attention upon milk and upon its fatty constituent. In this connection McCollum's statement[15] may be quoted that " an examination of any large group of people in the cities will show that where there is a high mortality from tuberculosis, milk is not being used to any great extent, and in any large group where milk purchases are large, the disease is not a menace. . . . There can be no reasonable doubt that the importance of poor hygienic conditions and of poor ventilation have been greatly overestimated, and that of poor diet not at all adequately appreciated as factors in promoting the spread of this disease. . . . Milk is our greatest protective food, and its use must be increased."

**Milk.**

Adherents of the theory of immunisation and of the theory of natural resistance may each claim support for their views if the conclusion pointed to, namely, that increased consumption of milk and butter has been closely associated with the decline in the phthisis death-rate, be considered worthy of credence. The former may claim that a proportion of all milk contains bovine tubercle, and that increased ingestion of this germ may result in a species of vaccination against human tubercle. Certainly in those districts where there is a large number of deaths from tuberculosis in children, *i.e.*, at the age when bovine tuberculosis is most prevalent, the disease is less common in the " young adult " than in other districts[17] ; this fact may be claimed as evidence of the young adult being somewhat immunised, while the occurrence of more tuberculosis than normal in " middle age " in the same districts may be claimed as evidence of this immunity wearing out with advancing years. On the other hand, there is no clear evidence that bovine tuberculosis does in fact protect against the human variety ; and the finding of both strains of the disease simultaneously in the same person is distinctly against the supposition. In Guernsey and Jersey, where tuberculosis has been stamped out among cattle, human tuberculosis is neither abnormally high nor abnormally low. In India, where tuberculosis is practically absent among cattle, human tuberculosis has long been endemic and would appear to be on the increase (possibly due to the influences previously discussed connected with the spread of modern civilisation and improved transport) ; unfortunately reliable statistics are lacking with regard to the incidence of the disease[18]. In that country the usual manifestations of the disease when of bovine origin, *i.e.*, tuberculosis of the joints and glands, are reported not to occur ; but no significance attaches to this fact, since the national custom has long been to boil all milk before it is consumed.

The evidence already given that more milk is being consumed in this country than formerly receives confirmation from the mortality data of Table VIII. ; here the only form of tuberculosis which has not declined in the last 50 years is found to be that wherein are included tuberculosis of bones and joints, *i.e.*, the group which contains the largest

Table VIII.—Annual Mortality from Different Forms of Tuberculosis.

| Sex. | Years. | Pulmonary tuberculosis and Phthisis. 0–5 | Pulmonary tuberculosis and Phthisis. All ages. | Meningitis. | Peritonitis. | All other forms (including bones and joints). |
|---|---|---|---|---|---|---|
| Males ... ... | 1861–70 ... | 994 | 2612 | 345 | 271 | 129 |
| | 1901–10 ... | 351 | 1358 | 189 | 160 | 184 |
| *Percentage rise or fall* | ... ... | +64·7 | +48·0 | +45·2 | +41·0 | −42·6 |
| Females ... ... | 1861–70 ... | 951 | 2578 | 253 | 243 | 103 |
| | 1901–10 ... | 304 | 951 | 172 | 145 | 156 |
| *Percentage rise or fall* | ... ... | +68·0 | +63·2 | +32·0 | +40·3 | −51·4 |

proportion of bovine cases. At the same time, other forms of the disease, meningitis and peritonitis, which especially affect childhood, have fallen in much the same way that pulmonary tuberculosis has fallen; while pulmonary tuberculosis under the age of five (most of which occurs during the first year of life, before bovine tuberculosis could exert any protective influence) has fallen more than at later years.

The evidence on the whole is distinctly against milk exerting upon tuberculosis as a whole any beneficial influence it may possess through the agency of bovine tuberculosis. Moreover, we have seen reason to hold that tuberculosis does not establish in any pronounced way an immunity against itself; therefore, on theoretical grounds at any rate, another allied germ, such as bovine tuberculosis, could hardly be expected to act in this way.

Those who hold the theory that milk supply may have exerted an influence by building up a metabolism "proper" for resisting tubercular infection may find support in the fact that "milk, cream, butter, cod-liver oil, all rich sources of the food factor, fat—soluble A, form the basis of the treatment of diseases of malnutrition and of tuberculosis, and it must be admitted that such foodstuffs are more than mere sources of fat, otherwise the cheaper fats, such as lard and the vegetable oils, would long ago have been adopted as equally efficient for the purpose."[16]

Diagram 5.—Comparison between Rise in Consumption of Milk and Butter and Fall in Phthisis Mortality.

Deduction.—The conclusion of the matter seems to be that undoubtedly a pure milk supply would eradicate bovine tuberculosis. And there is some probability that an increased consumption would beneficially affect the prevalence of tuberculosis. Hence every effort should be made to increase the supply and to ensure its purity.

**Segregation.**

There is not at present available sure evidence that segregation of tuberculosis is a potent weapon for controlling the disease. Probability suggests it. If the decline which has been taking place continues, the accommodation at present provided in Sanatoria should ere long be sufficient to meet any campaign directed to this end.

**Summary.**

Tuberculosis is an endemic disease which does not occur in epidemics; it runs no definite clinical course; and its prevalence varies with that of the general death rate. In these respects it differs from those infectious diseases, attacks of which confer immunity against further attack. It has been declining in prevalence as general wealth has been increasing, and is most prevalent where poverty is most pronounced. Evidence points to underfeeding being the influence associated with poverty which affects the disease; and there is some evidence that an important food in this connection is milk, and that possibly its fatty constituent is especially concerned. The only form of tuberculosis which has not declined is that due to the bovine strain of the bacillus. The tentative conclusion to be drawn is that a cheap and pure milk supply might further reduce the incidence of the disease so as to bring it under control.

References.

(12) Collis, E. L. "Tuberculosis Considered with regard to the Conditions of Factory Life." Trans. Sixth Ann. Confer. Nation. Assoc. for Prev. of Consump. Leeds. 1914.

(13) Greenwood, M. "The Epidemiology of Pulmonary Tuberculosis." Appendix V. Annual Report of the Chief Medical Officer, Ministry of Health. 1920. H.M. Stationery Office. (Cmd. 978).

(14) Vernon, H. M. "Industrial Efficiency and Fatigue." 1921. G. Routledge & Sons, Ltd.

(15) McCollum. "The New Knowledge of Nutrition," p. 152. 1919. Macmillan Publishing Co.

(16) "The Present State of Knowledge Concerning Accessory Food Factors (Vitamins)." Report No. 38. Medical Research Committee, 1919.

(17) Brownlee, J. "An Investigation into the Epidemiology of Phthisis in Great Britain and Ireland." Part III. Report No. 46. Medical Research Committee, 1920.

(18) Lankester, A. "Tuberculosis in India." 1920. Butterworth & Co.

### EX-SERVICE MEN IN SANATORIA.

The Minister of Health, in consultation with the Treasury and the Ministry of Pensions, has made arrangements for the concurrent treatment and vocational training of ex-Service men at the following sanatoria :—

(1) Market gardening, poultry, pig and bee keeping: Burrow Hill Sanatorium, Frimley; pig and poultry keeping: Middleton Sanatorium, Ilkley.

(2) Rural carpentry: Burrow Hill Sanatorium, Frimley; Middleton Sanatorium, Ilkley; the National Sanatorium, Benenden, Kent; Liverpool Sanatorium, Kingswood.

(3) Furniture repairing: Holywood Hall Sanatorium, Wolsingham, Durham; Lenham Sanatorium, Lenham, Kent; the Borough Sanatorium, Leicester; Maltings Farm Sanatorium, Nayland; West Heath Sanatorium, Birmingham; Middleton Sanatorium, Ilkley.

(4) House repairs: Liverpool Sanatorium, Kingswood; Holywood Hall Sanatorium, Wolsingham; Lenham Sanatorium, Lenham, Kent; Crooksbury Sanatorium, near Farnham, Surrey; Burrow Hill Sanatorium, Frimley; West Heath Sanatorium, Birmingham.

(5) Tin smithing, art metal work, &c.: West Heath Sanatorium, Birmingham.

(6) Brush and basket making: The National Sanatorium, Benenden, Kent.

(7) Jewellery, watch, clock and china repairing: Liverpool Sanatorium, Kingswood; Maltings Farm Sanatorium, Nayland.

These courses have been designed to give men desiring to work on their own account in suburbs, country towns or large villages a general training which should enable them to earn a living. It is not anticipated that the training given will make men commercially successful at once, but it will teach the necessary principles and will give them such knowledge that commercial success should follow with experience. Particulars as to applications for admission to the courses are contained in a circular issued by the Ministry of Pensions. Payment for the treatment and training will be made by the Ministry of Health direct to the authorities of the institution in which the training is provided.

### THE ROYAL INSTITUTE OF PUBLIC HEALTH.

At the Annual Congress of the Royal Institute of Public Health at Plymouth, on May 31—June 5, Section IV is devoted to the discussion of "Women and Public Health," and in view of the very great interest which the subject holds at the present time, the Council of the Institute offers special facilities to women health officials, district nurses, voluntary infant welfare workers, midwives, welfare workers and other social workers to enable them to attend the two days' discussion at a special fee of five shillings; full membership of the Congress, one guinea. Tickets can be obtained from the Royal Institute of Public Health, 37, Russell Square, London, W.C.2.

The meetings of this Section will be held in the Council Chamber, Municipal Buildings. On Thursday, June 1, papers will be read on various aspects of maternity work (morning) and infant welfare work (afternoon); on Friday, June 2, the subjects will be "The School Child" (morning) and "Racial and Industrial Aspects of Maternity" (afternoon).

Delegates and others attending the Congress are advised to secure accommodation without delay, as the meeting coincides with the Bath and West of England Agricultural Show in Plymouth.

---

We are asked by the Church Missionary Society, Salisbury Square, London, E.C.4, to state that at the present moment it is in urgent need of the following workers in order to bring up its mission hospital staffs to the minimum essential for efficiency. Further particulars may be obtained direct from the Society :—

| | Doctors. | | | |
|---|---|---|---|---|
| | Men. | Women. | Nurses. | Chemists. |
| West Africa ... ... | 1 | — | 1 | — |
| East Africa ... ... | 4 | — | 3 | — |
| Uganda ... ... | 1 | — | 1 | — |
| Egypt and Sudan ... | — | — | 4 | — |
| Palestine ... ... | 1 | 2 | — | — |
| Persia ... ... ... | 3 | 1 | 3 | — |
| India ... ... ... | 4 | 2 | 5 | 1 |
| China ... ... ... | 11 | 1 | 8 | 1 |
| | 25 | 6 | 25 | 2 |

## Figures and Tables

**Diagram 4. f1:**
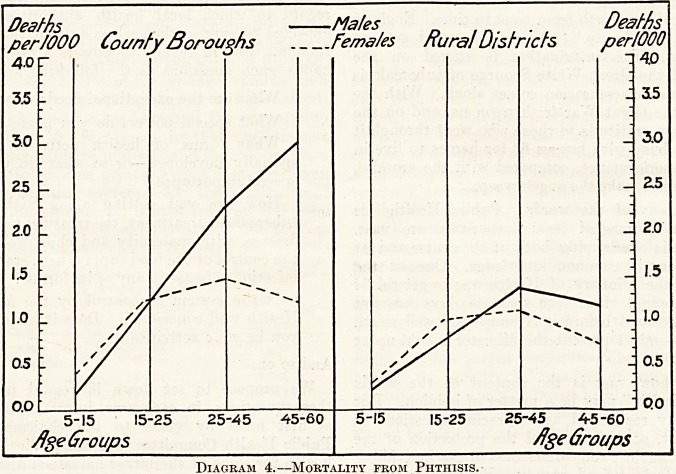


**Diagram 5. f2:**